# Do not throw out the baby with the bath water: choosing an effective baseline for a functional localizer of speech processing

**DOI:** 10.1002/brb3.129

**Published:** 2013-02-17

**Authors:** Nadav Stoppelman, Tamar Harpaz, Michal Ben-Shachar

**Affiliations:** 1 The Gonda Multidisciplinary Brain Research Center, Bar Ilan UniversityRamat Gan, Israel; 2 Linguistics Division, English Department, Bar Ilan UniversityRamat Gan, Israel

**Keywords:** fMRI, functional localizer, reversed speech, signal correlated noise, speech perception

## Abstract

Speech processing engages multiple cortical regions in the temporal, parietal, and frontal lobes. Isolating speech-sensitive cortex in individual participants is of major clinical and scientific importance. This task is complicated by the fact that responses to sensory and linguistic aspects of speech are tightly packed within the posterior superior temporal cortex. In functional magnetic resonance imaging (fMRI), various baseline conditions are typically used in order to isolate speech-specific from basic auditory responses. Using a short, continuous sampling paradigm, we show that reversed (“backward”) speech, a commonly used auditory baseline for speech processing, removes much of the speech responses in frontal and temporal language regions of adult individuals. On the other hand, signal correlated noise (SCN) serves as an effective baseline for removing primary auditory responses while maintaining strong signals in the same language regions. We show that the response to reversed speech in left inferior frontal gyrus decays significantly faster than the response to speech, thus suggesting that this response reflects bottom-up activation of speech analysis followed up by top-down attenuation once the signal is classified as nonspeech. The results overall favor SCN as an auditory baseline for speech processing.

## Introduction

Speech processing is a multistage operation that engages several cortical regions in the temporal, parietal, and frontal lobes. Evidence from anatomical and functional neuroimaging studies supports the view that speech is processed along hierarchically organized streams (Scott and Johnsrude 2003; Hickok and Poeppel 2007; Davis et al. 2011; Lerner et al. 2011). According to this view, auditory aspects of speech are processed in and around the core of the auditory cortex, while the processing of high-level linguistic features extends into posterior, lateral, anterior, and inferior temporal regions as well as inferior frontal regions (Belin et al. 2000; Binder et al. 2000; Scott et al. 2000; Davis and Johnsrude 2003; Narain et al. 2003; Rodd et al. 2005; Andics et al. 2010; DeWitt and Rauschecker 2012). Localization of such language-sensitive regions in individual brains is important for both research and clinical purposes, for example, when studying subtle linguistic contrasts (Ben-Shachar et al. 2003, 2004), developmental populations (Wilke et al. 2006; Rauschecker et al. 2009; Ben-Shachar et al. 2011), and in presurgical mapping (Swanson et al. 2007; Chakraborty and McEvoy 2008; Kipervasser et al. 2008; Bick et al. 2011).

Localizing speech responses in an individual participant using functional magnetic resonance imaging (fMRI) is complicated by several factors. First, particularly along superior temporal regions, cortical responses to sensory and linguistic aspects of speech are tightly packed, making it difficult to isolate responses to linguistic aspects of speech from primary auditory responses (Scott and Johnsrude 2003). Delineating language responses according to anatomical markers is further complicated by known individual variability in the mapping between cytoarchitectonic areas and gross anatomy (Amunts et al. 2000; Rademacher et al. 2001). An effective solution to these problems is to use a functional localizer to isolate speech-specific responses, by contrasting speech responses against responses to an auditory baseline. In this article, we discuss the considerations in choosing such a baseline, and compare the localizing value of two widely used baselines for auditory speech processing.

A functional localizer is a short fMRI scan which is added to the scan protocol in order to identify the individual's regions of interest (ROIs) (Fedorenko et al. 2010; Saxe et al. 2006). For example, in the visual domain, ROIs such as V1, V2, hV4, and so on are typically identified in individual participants using retinotopy scans (Engel et al. 1994). Similarly, regions sensitive to visual faces and words are often localized by contrasting face versus house stimuli and words versus checkerboards, respectively (Kanwisher et al. 1997; Cohen et al. 2000; Duncan et al. 2009). In the context of speech processing, an optimal functional localizer aims to satisfy the following constraints: (a) Efficiency: Short scan, about 3–5 min long. This is most important in developmental and clinical populations; (b) Sensitivity: Evoke robust BOLD signals in each person's speech-selective regions to allow ROI definition at the individual level; (c) Specificity: Isolate speech responses from other sensory and cognitive components. Optimally, this is achieved by contrasting speech responses with a control stimulus that has exactly the same *acoustic* properties as speech, without any of its *linguistic* properties; (d) Independence: Functional localizers should be general enough to be considered independent of the effect of interest in as much as possible. For example, if the main experiment contrasts passive versus active sentences, the localizer should not include a large ratio of passive sentences. This is important in order to avoid “double dipping” or selection bias in the population of voxels identified by the localizer (Vul et al. 2009).

To satisfy the efficiency and sensitivity requirements, localizers are typically conducted in a block design. This means that several stimuli of the same condition are presented sequentially to enhance the BOLD signal in an additive manner, thus increasing sensitivity. A block design also presents with maximal efficiency (Dale 1999). However, satisfying the specificity requirement in its strong form (as stated in c) is logically impossible if one considers phonology and prosody as linguistic properties, as they are acoustically defined.

An empirical approach to this problem is to look for a baseline that controls for sensory responses as much as possible without losing the speech signal in temporal and frontal language regions. Since the emergence of functional neuroimaging, speech perception researchers and clinicians have used a wide array of baseline conditions which were thought to satisfy these criteria. These include foreign language (Perani et al. 1996), pseudowords (Binder et al. 1994), reversed speech (Price et al. 1996), signal correlated noise (SCN) (Rodd et al. 2005), spectrally rotated speech (Scott et al. 2000), or music (Bleich-Cohen et al. 2009). Recently, Binder et al. (2008) compared five fMRI protocols for mapping the speech processing network, demonstrating that the choice of baseline is critical for clinical mapping. However, their analysis focused on group-level comparisons, so it is hard to deduce which protocol will be the most advantageous as a functional localizer at the individual subject level. Here, we chose to focus on two distinctively popular baselines: reversed speech and SCN. Our main goal is to provide an empirical test of how well they do in achieving the sensitivity and specificity criteria described above, at the individual subject level.

Reversed speech is a control stimulus that enjoys high popularity in functional imaging setups (Perani et al. 1996; Price et al. 1996; Dehaene et al. 1997; Hirano et al. 1997; Wong et al. 1999; Binder et al. 2000; Dehaene-Lambertz et al. 2002; Crinion et al. 2003; Crinion and Price 2005; Leff et al. 2008; Redcay et al. 2008; Strand et al. 2008; Warren et al. 2009). Reversing speech is technically simple (e.g., in Matlab, sound(flipud(y),Fs) will play y backward at Fs sampling frequency). This temporal reversal results in an unintelligible stimulus that matches the original in its global acoustic characteristics, including division into words, voicing, and some articulatory features (e.g., fricatives). Crucially, as the temporal envelope of the original speech is reversed, the manipulation breaks much of the phonotactic structure of speech, as well as phrase and sentence level prosody (Narain et al. 2003).

Another stimulus that has become increasingly common in recent studies of speech perception is SCN (Mummery et al. 1999; Rodd et al. 2005; Coleman et al. 2007; Davis et al. 2007; Little et al. 2010; Peelle et al. 2010; Zheng et al. 2010; Travis et al. 2011). SCN is created by replacing all the spectral detail in the original speech stimulus with noise, while maintaining the envelope of the original waveform (Schroeder 1968). Paragraphs processed in this manner retain speech-like rhythmic onsets, but they do not control for other features of speech (e.g., pitch, phonemic structure).

We contrasted listening to Hebrew speech against these two baselines, reversed speech and SCN. As far as we know, this is the first study to compare the efficacy of these commonly used baseline conditions in localizing the core language areas of individual subjects. In particular, we compared the efficacy of each of these baselines in removing responses in primary auditory cortex, and in retaining responses in known frontal and temporal speech processing regions. We further examined the temporal profile of the responses to different stimulus conditions within frontal and temporal regions. The results point to similar specificity of both baselines around primary auditory cortex, but a clear sensitivity advantage for the baseline of SCN in inferior frontal cortex.

## Methods

### Subjects

Participants were twelve healthy adult volunteers (seven females, mean age 27.3 ± 4). All were native speakers of Hebrew, without any history of hearing or language impairment. All participants were strongly right handed (70% or higher in the Edinburgh Handedness Inventory; Oldfield 1971). All of them gave informed consent to participate in the study, in accordance with a protocol approved by the Helsinki Committee of Tel Aviv Sourasky Medical Center.

### Stimuli

Four short speech epochs were recorded in Hebrew by a female native speaker in a silent chamber. We used excerpts from children's poems, suitable for a wide age range including young children (Gefen 1974; Atlas 1977). The recorded segments, each lasting 15 sec, were digitized at a sampling rate of 44 kHz, and scaled to an average intensity of 75 dB.

Using Praat software (http://www.praat.org), we applied two forms of distortion to these paragraphs, resulting in two unintelligible baseline conditions. Both baselines largely preserve aspects of the spectral profile and amplitude envelope of the original speech stimulus, but their acoustic properties are markedly different. Example audio files are included as supplementary material.

#### Signal correlated noise

The SCN baseline was created by extracting the amplitude envelope of a speech segment and applying it to a pink noise segment, band-pass filtered to maintain the original frequency spectrum of speech. This resulted in an amplitude-modulated noise stimulus which preserved the amplitude variations and the spectral profile of the original speech. SCN stimuli were generated using Praat code (based on code from Matt Davis, MRC Cognition and Brain Sciences Unit, Cambridge).

#### Reversed speech

The reversed speech baseline was created by reversing the speech stimulus in time, as if it was played backwards from end to start.

### Procedure

In order to track the time-evolving response that reflects phrasal-level processing, we employed a continuous sampling paradigm (simultaneous scanning and stimulus presentation). While background noise may partially mask the auditory stimuli and reduce sensitivity somewhat, continuous sampling is still advantageous in that it enhances statis-tical power and shortens scan time significantly, simply by collecting more images per scan minute, and speeding up the stimulus presentation rate.

**Figure 1:**
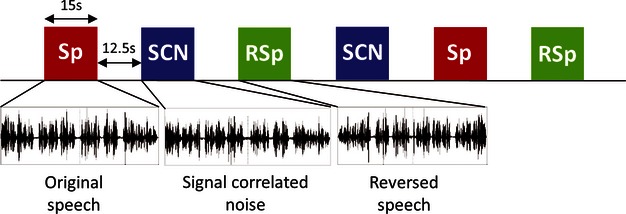
Experimental design. Schematic plot of a single experimental run. The experiment consisted of two runs, each containing a total of six blocks (interleaved with rest blocks), two blocks for each stimulus type. Block presentation was pseudorandomized, so that no two consecutive blocks were of the same condition. Sp (red), speech; SCN (blue), signal correlated noise; RSp (green), reversed speech.

Stimuli of three conditions, *Speech*, *Reversed,* and *SCN*, were presented in a simple block design, for the purpose of improving sensitivity in individual subjects. Blocks consisted of a single paragraph, 15 sec long, and were interleaved with 12.5 sec rest epochs (see Fig. 1). In order to ensure that subjects were paying attention during stimulus presentation, they performed an auditory detection task of auditory “blip” cues and responded with a button press (three cues randomly placed in each experimental block, scaled to the same intensity as the auditory stimuli). This orthogonal task allowed us to direct and monitor participant's attention to auditory stimuli of all conditions.

Before entering the scanner, subjects underwent a brief training session in order to get familiarized with the task and the different stimulus types. Participants were instructed to maintain their gaze on a central fixation cross which appeared throughout the entire experiment, listen attentively to all auditory stimuli, and respond when they hear the target cue. E-Prime 2.0 (Psychology Software Tools, Pittsburgh, PA) was used for stimulus presentation and response collection. Stimuli were delivered to the subjects via MR compatible headphones, which are part of a customized recording system (FOMRI-III; Optoacoustics, Israel) implementing active noise cancelation. A short auditory test was delivered during scanning to confirm that subjects could hear the stimuli clearly above the scanner noise. The experiment was divided into two short runs (3:20 min long each), separated by a short break. A single run consisted of six experimental blocks (interleaved with rest blocks), with two blocks of each of the three conditions randomized so that no two consecutive blocks were of the same type (Fig. 1).

### Data acquisition

fMRI data were collected on a GE 3T scanner located at the Wohl Institute for Advanced Imaging at Tel Aviv Sourasky Medical Center. Thirty-two functional (T2* weighted) and anatomical (T1 weighted) oblique slice were acquired along the ac-pc plane (3 mm thick, no gap), covering the whole temporal lobe and most of the frontal lobe (TR = 2500 msec, TE = 35 msec, flip angle = 90°, voxel size = 2.3 × 2.3 × 3 mm). In addition, high-resolution anatomical images were acquired for each subject using fast spoiled gradient echo (SPGR) sequence. In-plane anatomical images were used to align the functional data with the high-resolution anatomical data, allowing volume-based statistical analyses of signal changes along time.

### Data analysis

fMRI data were preprocessed, analyzed, and visualized using MATLAB (The Mathworks, Nattick, MA) and mrVista tools (http://white.stanford.edu/software). Individual subject analyses were applied at native space of each participant, without spatial smoothing, in order to maintain the high spatial resolution provided by MRI. The first five fMRI volume images of each run were excluded from analysis to ensure steady-state magnetization. General linear model (GLM) predictors were constructed to estimate the relative contribution of each condition to every voxel's time course, using a boxcar function convolved with a canonical hemodynamic response function (HRF). Spatial contrast maps were computed for each contrast of interest, based on voxel-wise *t*-tests between the weights of relevant predictors. Functional ROIs were selected by marking continuous clusters of voxels that passed the threshold of *p <* 10^−3^ (uncorrected) within anatomically defined borders, as detailed below. This threshold was equivalent to a false discovery rate (FDR) corrected value of *q* < 0.1. ROIs were defined in left inferior frontal gyrus (LIFG), bilateral posterior superior temporal sulcus (pSTS), and bilateral anterior superior temporal sulcus (aSTS). The decision to focus on this particular set of ROIs was guided by numerous preceding studies of speech processing and current models of speech processing (e.g., Scott and Johnsrude 2003; Price et al. 2005; Friederici 2011 among many others), and by a general inspection of the individual data confirming the existence of consistent activation in these areas for speech versus rest. The anatomical borders of the ROIs were defined as follows: (a) IFG: pars opercularis and pars triangularis of the IFG; (b) pSTS: the posterior third of the superior temporal sulcus, including BA 39 bordering BA 37, BA 22; (c) aSTS: the anterior third of the STS, including BA 38 and the anterior part of BA 22, bordering BA 21. Mean cluster size was calculated by averaging the volumes of activated voxels within an ROI across all participants, considering null activation as zero.

Time course data were collected from ROI voxels identified by *Speech* versus *SCN* contrast in the native in-plane slices to avoid smoothing and interpolation. Drawing on results from the previous analyses, our goal was to compare specifically between the time courses of speech and reversed speech. Mean time course of the BOLD signal was calculated by averaging the responses to each condition across the four repetitions. We then computed the half-maximum decay time as the time lag from the block onset to the time when the activation reached half of the peak value (we used linear interpolation to extract this time point, because in most cases the response reached half the maximum in between samples).

## Results

Participants performed the auditory detection task easily and with high accuracy (>90%) providing confirmation of attention maintenance throughout the experiment.

In order to compare the efficacy of the two baselines (SCN, Reversed), we first calculated the likelihood of detecting significant activation in the language network per individual using each baseline. The identification rate of core regions of the speech processing network (LIFG, bilateral pSTS, bilateral aSTS) was significantly higher in the *Speech* versus *SCN* contrast (93%) than in the *Speech* versus *Reversed* contrast (55%) (*χ*^*2*^ (1,59) = 20.58; *p* < 0.0001) (see Table 1).

**Table 1:** Identification rate of core speech processing regions

ROI	Baseline	# of subjects (*n* = 12)	*X*	*Y*	*Z*
Left IFG	Reversed	3	−50 ± 6	17 ± 12	18 ± 11
	SCN	11	−45 ± 7	23 ± 9	13 ± 12
Left pSTS	Reversed	10	−56 ± 9	−36 ± 10	4 ± 3
	SCN	12	−58 ± 9	−36 ± 7	5 ± 5
Right pSTS	Reversed	7	52 ± 11	−37 ± 7	8 ± 2
	SCN	12	50 ± 10	−35 ± 8	7 ± 4
Left aSTS	Reversed	7	−56 ± 3	−1 ± 6	−13 ± 7
	SCN	10	−56 ± 2	−2 ± 6	−8 ± 6
Right aSTS	Reversed	6	58 ± 3	−1 ± 9	−12 ± 5
	SCN	11	57 ± 2	1 ± 9	−10 ± 5

Number of participants showing significant clusters of activation (cluster size larger than 70 mm^3^ at *P* < 0.001, uncorrected) in each core component of the speech processing system. Identification rate is reported for the contrast of *Speech* versus *Signal Correlated Noise* (SCN) and *Speech* versus *Reversed speech* (Reversed). SCN baseline proved more successful in localizing each of the speech-related ROIs. The three rightmost columns show mean MNI coordinates (±SD) of the center of mass of the ROIs defined using each baseline. Overall, both contrasts define similar anatomical locations with reduced sensitivity using the reversed speech baseline.

**Figure 2:**
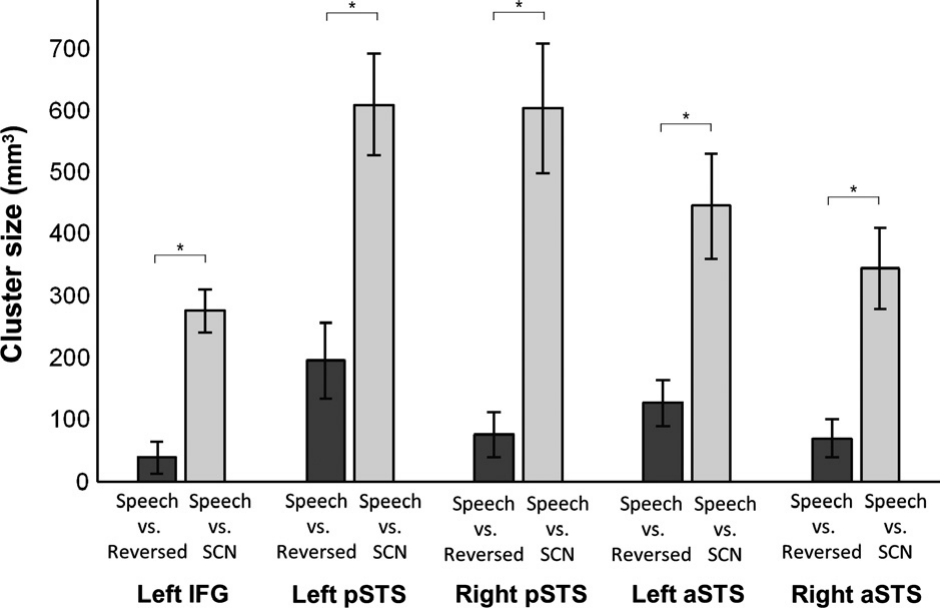
Cluster size comparison. Clusters were defined by contrasting *Speech* versus *Reversed* (dark gray) and *Speech* versus *SCN* (light gray), within the anatomical boundaries of Left IFG, bilateral pSTS, and bilateral aSTS (all defined individually at a threshold of *P* < 0.001, uncorrected). *Speech* versus *SCN* yields significantly larger clusters across the five ROIs (*F* (1,11) = 63.8; *P* < 0.001). SCN, signal correlated noise; IFG, inferior frontal gyrus; pSTS, posterior superior temporal sulcus; aSTS, anterior superior temporal sulcus; ROIs, regions of interests.

The above analysis considers each ROI as an all-or-none value (activation passes the threshold or not). To further quantify the difference between the two baselines, we compared the mean cluster size for each contrast across all anatomical locations (Fig. 2). An analysis of variance (ANOVA) produced a significant main effect of baseline condition (*F* (1,11) = 63.8; *p* < 0.001), with larger clusters elicited by the *Speech* versus *SCN* contrast compared with the *Speech* versus *Reversed* contrast (mean volumes: 452 mm^3^ and 101 mm^3^, respectively). Post hoc *t*-tests confirmed that the *Speech* versus *SCN* contrast elicited larger clusters of activation in each region (*p* < 0.001). We also observed a significant main effect of ROI location (*F* (4,44) = 5.3; *p* < 0.002), reflecting larger clusters in bilateral posterior and anterior STS compared with LIFG across baselines. Finally, we observed a significant interaction between baseline condition and ROI location (*F* (4,44) = 4.2; *p* < 0.006), revealing a more pronounced cluster size difference between the *Speech* versus *SCN* contrast and the *Speech* versus *Reversed* contrast in bilateral pSTS regions.

**Figure 3:**
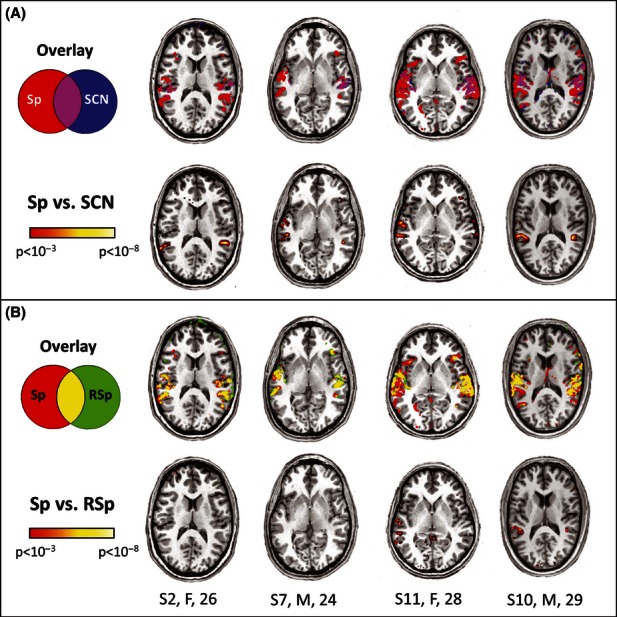
Overlay maps in posterior superior temporal cortex. Axial slices of four individual participants depicting significant responses for each contrast centered on bilateral posterior superior temporal cortex (*P* < 0.001, uncorrected). (A) Overlay of binary activation maps for *Speech* versus *Rest* (red) and *SCN* versus *Rest* (blue), followed by the direct contrast map for *Speech* versus *SCN* in the same participants. SCN overlaps with Speech mostly in primary auditory cortex (magenta patches) but not in more posterior temporal regions. (B) Overlay of binary activation maps for *Speech* versus *Rest* (red) and *Reversed* versus *Rest* (green), followed by the direct contrast map for *Speech* versus *Reversed*. Notice the reduced temporal signals in the *Speech* versus *Reversed* contrast, stemming from the largely overlapping patterns of activation for speech and reversed (yellow patches). Sp, speech; SCN, signal correlated noise; RSp, reversed speech.

Next we calculated, for each subject, the overlay between speech and each of the baseline conditions, as well as contrast maps that directly compare the spatial distribution of signals using each baseline condition. Figure 3 shows such overlay maps in four individual participants centered on bilateral pSTS. These representative maps demonstrate best the overall findings. As can be seen in Figure 3A, both speech and SCN activated Heschl's complex (appearing in magenta in the overlay map, Fig. 3A, top panel), but only speech activated surrounding temporal areas (appearing in red in Fig. 3A). Accordingly, activation in Heschl's complex, but not in pSTS, was selectively removed in the direct contrast *Speech* versus *SCN* (Fig. 3A, bottom panel). In comparison, the reversed speech baseline produced activation patterns that overlap heavily with the speech activation pattern in extended parts of the superior temporal cortex, as shown in the extended magenta-colored areas in Figure 3B (top panel). Thus, reversed speech successfully eliminates activation in Heschl's complex, but, at the same time, reduces activation in the pSTS in the direct contrast *Speech* versus *Reversed* (Fig. 3B, bottom panel) and sometimes eliminates it altogether (S2, S7).

**Figure 4:**
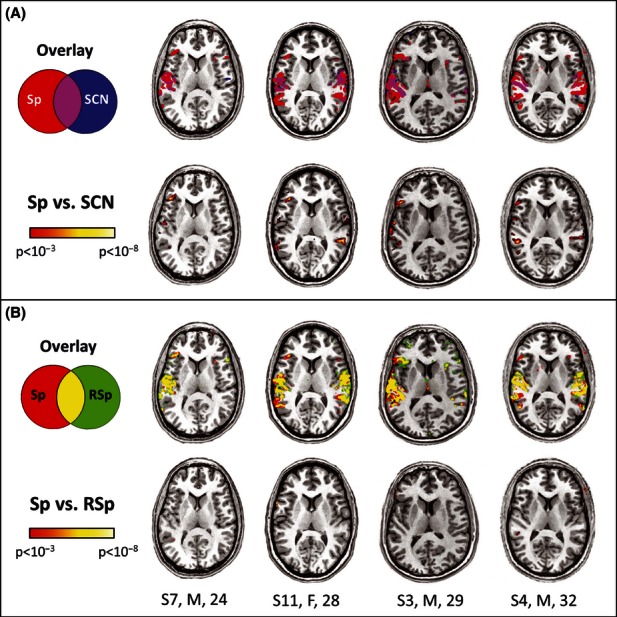
Overlay maps in left inferior frontal gyrus. Axial slices of four individual participants depicting significant responses for each contrast in the left IFG (*P* < 0.001, uncorrected). Same conventions and color schemes as in Figure 3. (A) Overlay of binary activation maps for *Speech* versus *Rest* and *SCN* versus *Rest*, followed by the direct contrast map for *Speech* versus *SCN* in the same participants. (B) Overlay of binary activation maps for *Speech* versus *Rest* and *Reversed* versus *Rest*, followed by the direct contrast map for *Speech* versus *Reversed* (notice scarcity of activated voxels). IFG, inferior frontal gyrus; SCN, signal correlated noise.

Similar maps are demonstrated in Figure 4, this time centered on the left IFG. In each of these subjects, speech, but not SCN, consistently activated the left IFG (Fig. 4A, top panel). Consequently, SCN successfully retained frontal activations in the direct contrast *Speech* versus *SCN* (Fig. 4A, bottom panel). Reversed speech, on the other hand, exhibits activation patterns that overlap considerably with speech in left IFG, as denoted in yellow in this area (Fig. 4B, top panel). These overlapping patterns result in the removal of left IFG activation in the direct contrast *Speech* versus *Reversed* (Fig. 4B, bottom panel). Comparisons in bilateral aSTS exhibited similar overlap patterns as in the pSTS (not shown). Hence, our findings suggest that reversed speech is suboptimal as a baseline for speech localization, possibly because language regions attempt to parse it as linguistic input.

**Figure 5:**
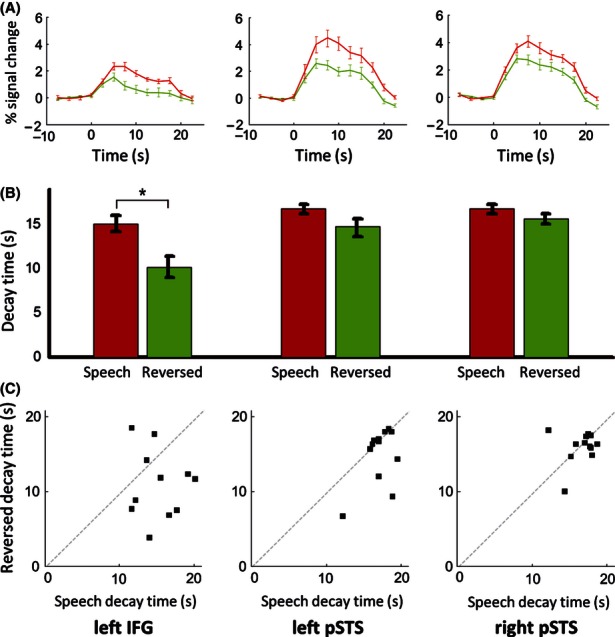
LIFG responses to reversed speech decay faster than the response to speech. (A) Group-averaged time course of BOLD activation for speech (red) and reversed speech (green) in three functionally defined ROIs. ROIs were defined by *Speech* versus *SCN* (*P* < 0.001, uncorrected). Time = 0 denotes block onset. (B) Half-maximum decay time of the BOLD response for speech and reversed speech. Bars denote group averages, error bars represent 1 standard error of the mean. Signal decay is significantly faster for reversed speech than speech in LIFG (*n* = 11, *t* (2,20) = 2.53, *P* < 0.05, Bonferroni corrected for multiple comparisons across the five ROIs). (C) Half-maximum decay times are plotted for speech against reversed speech in each participant. Dots under the gray line (*x* > *y*) are ones where reversed speech decays faster than speech. This happens in the majority of subjects in LIFG, but only in a handful of subjects in LpSTS. LIFG, left inferior frontal gyrus; ROIs, regions of interests; SCN, signal correlated noise; pSTS, posterior superior temporal sulcus.

To better characterize the similarities and differences in BOLD responses to speech and reversed speech, we examined the time courses to each of these conditions within core speech-sensitive regions. We found that both speech and reversed speech indeed activate these regions, with some advantage for the speech condition (Fig. 5A). Importantly, this advantage was evident in all three ROIs independently of the contrast used to define the ROIs (both using the contrast of *Speech* vs. *SCN* and using the contrast *Speech + Reversed* vs. *Rest*). We also noticed a more subtle difference between the temporal profiles of these responses in LIFG: the response to reversed speech rises together with the response to speech, but decays faster. This effect is seen more clearly in the individual time courses (Fig. S3). We quantify this effect by calculating the half-maximum decay time of the BOLD response for speech and reversed speech, in each of the ROIs. Note that we did not include the SCN responses in this analysis because they did not show a clear peak in these regions, and so an analysis of half-maximum decay time would simply pick up noise fluctuations.

The analysis of half-maximum decay times (Fig. 5B) reveals that in left IFG, but not in temporal ROIs, the response to reversed speech decays significantly faster than the response to speech (*t* (2,20) = 2.53, *P* < 0.05, Bonferroni corrected for multiple comparisons across the five ROIs, Fig. 5B). Time course results for bilateral aSTS were qualitatively similar to those found in bilateral pSTS (Fig. S1). We repeated the analysis using an orthogonal contrast (*Speech + Reversed* vs. *Rest)* and replicated the decay time effect in left IFG (*t* (2,20) = 2.77, *P* < 0.05, not shown), verifying that the effect remains significant regardless of ROI definition. This effect was seen in the majority of our participants (eight out of eleven, Fig. 5C), suggesting that LIFG initially attempts to analyze reversed speech as linguistic input, but gives up once this input is recognized as nonspeech.

## Discussion

We compared two auditory baselines commonly used in functional localizers of speech processing, reversed speech and SCN. While both baselines adequately remove activation in primary auditory cortex, reversed speech removed much of the activation in language regions as well. This effect is detrimental particularly in the left IFG, where only 3 out of 12 participants showed activated clusters for *Speech* versus *Reversed*, compared with 11 participants in the *Speech* versus *SCN* contrast. This outcome is not threshold specific (see Fig. S2) but can be directly attributed to robust overlap between speech and reversed speech responses across the entire speech processing network, predominantly in the left IFG. A closer look at the time course and decay parameters of individual participants (Figs. S3 and 5C) provides a possible explanation to this effect: activation in LIFG rises similarly in the speech and reversed conditions, but then decays faster in the reversed condition. This suggests that LIFG attempts to parse reversed speech but then attenuates its response once the input has been recognized as nonlinguistic.

Our results have clear practical implications for both clinical and research applications of functional localizers of speech. In the clinical domain (e.g., presurgical mapping of speech regions in individual patients), false negatives could have irreversible consequences: a region which is not activated in the language localizer may be severed during surgery. On the basis of the significantly higher detection rate and cluster sizes documented here using SCN, and assuming that these results generalize to patient populations, we conclude that SCN is a better baseline for speech in clinical setups. This advantage may be enhanced when scanner noise increases. If we attribute the responses to reversed speech as unsuccessful attempts to parse it, we predict that the difference between baselines will be even more pronounced as scanner noise increases, that is, using high-field MRI and lower audio/headphone quality. Under such conditions, it could take longer to recognize that reversed speech is not speech, which will lengthen the overlap period between these responses. Importantly, providing a quiet epoch for stimulus presentation using sparse sampling or clustered acquisition is expected to improve the quality of the auditory stimulation and may thus reduce the advantage of SCN over reversed speech. Yet, sparse sampling requires prolonged acquisition time, and is typically used with event-related designs. These choices have their own disadvantages in the context of a functional localizer, particularly reduced power at the individual subject level and less efficient use of scan time (Dale 1999). Finally, SCN is preferred over a rest baseline if one aims to calculate lateralization indices in temporal speech processing regions, which are difficult to disentangle adequately from bilateral primary auditory responses without an active auditory baseline.

In basic research designs, functional localizers provide a tool for isolating language regions in individual participants, followed by an in depth analysis of the responses for well matched conditions in independent experiments within these ROIs. We have argued in the introduction that such a localizer should satisfy several constraints: efficiency, sensitivity, specificity, and independence (see also Fedorenko et al. 2010). On the basis of our results, we can now determine that reversed speech fails on sensitivity at the individual subject level. Low sensitivity at the individual level can be overcome in group analysis. Indeed, some fMRI studies report significant group activation maps for *Speech* versus *Reversed* (Crinion and Price 2005; Balsamo et al. 2006; Leff et al. 2008), though other group analyses have failed to do so (Binder et al. 2000; Ahmad et al. 2003). In a group analysis of the data reported here we still failed to detect activation for speech compared with reversed speech in the IFG (see Fig. S4).

We consider two alternative explanations for the inconsistency in group analyses of *Speech* versus *Reversed*: in terms of statistical power or in terms of the task manipulation. In our study, which targets individual localization of speech-related cortex, the small sample size (*N* = 12) may well have contributed to the null result achieved at the group level. A similarly small sample (of 15 children) has been used by Ahmad et al. (2003), and that study also fails to detect significant left IFG activation for stories versus reversed stories. A second relevant factor that may explain the variability in group results is task manipulation. It could be argued that the semantic content of speech must be explicitly attended in order to elicit left IFG activation. According to this explanation, lack of significant activation in language regions for *Speech* versus *Reversed* may have stemmed from our use of an orthogonal task (auditory cue detection), rather than a semantic task. Indeed, two fMRI studies that employed an explicit semantic task reported left IFG activation for words versus reversed words (Balsamo et al. 2006; Leff et al. 2008). In contrast, mixed findings are found with passive listening tasks: Significant IFG activation is found by Crinion and Price (2005), but not by Binder et al. (2000) and Ahmad et al. (2003), all applying group analyses of S*peech* versus *Reversed* under passive listening conditions. Taken together, these results suggest that by use of an active, semantic task one might enhance activation in core language regions for S*peech* versus *Reversed*.

The clear downside of using a semantic task in our localizer is that this task can only be performed on the speech condition, thus giving rise to a task by condition confound. Semantic tasks are also more complicated to perform by young subject populations, and are likely to cause performance differences between age groups. Using a simple auditory cue detection task, we satisfy the need to monitor individuals attention to all experimental stimuli (intelligible or not), in a way that is easy to perform by children and adults alike. As we show, there is a clear advantage for using SCN as baseline given this task choice.

Reversed speech (“backward speech”) is a popular baseline choice particularly in imaging studies of early development (Dehaene-Lambertz et al. 2002; Pena et al. 2003; Redcay et al. 2008). There is plenty of behavioral evidence that reversed speech can indeed be distinguished from speech at a very early age (Ramus et al. 2000; Pena et al. 2003). This ability likely relies on prosodic processing, rather than on speech comprehension which is not yet mature at this age group (Christophe et al. 2003). In agreement with this interpretation, Dehaene-Lambertz et al. (2002) found activation in right (not left) IFG for speech versus reversed speech in 3-month-old infants. In another study, Redcay et al. (2008) show bilateral frontal activations to speech versus rest in toddlers, but these activations disappear in the direct contrast speech versus reversed speech (Fig. 2). We propose that this result could point to positive responses to reversed speech in bilateral IFG, even at this very young age group. Reporting the responses to each condition separately (*Speech* vs. *Rest*; *Reversed speech* vs. *Rest*) as well as the direct contrast between them (as in Dehaene-Lambertz et al. 2002) is crucial in order to reach a proper interpretation of the effects in these young age groups.

While SCN is a better baseline for speech in terms of sensitivity, it is not flawless. A perfect baseline would be equated in all the acoustical features of speech, without sharing the linguistic features of speech. As some linguistic properties are defined acoustically (e.g., phonetic and prosodic aspects), a perfect baseline is impossible to achieve, leaving us with various compromises. Among the two alternative baselines compared here, SCN successfully removes primary auditory responses, but retains speech responses in frontal and temporal regions. When we use reversed speech as an auditory baseline in a continuous sampling paradigm, we risk “throwing out the baby with the bath water,” that is, removing too much of the signal in speech processing regions. An alternative approach to both of these localizers would target specific systems or processing pathways, via a more focused manipulation of syntax (cf. Fedorenko et al. 2010), morphology (Bick et al. 2008), and so forth. This approach could lead to a more refined identification of relevant ROIs. Importantly, such localizers should go through similar optimization procedures to allow maximum sensitivity, specificity, efficiency, and independence (see Fox et al. (2009) for a similar approach in a different domain). All in all, developing a set of standard, optimized, off-the-shelf localizers for specific language functions will allow better comparability across language studies and provide a systematic approach for single subject analyses in fMRI.

## Supporting Information

**Figure S1.** Temporal characteristics of a STS response to speech and reversed speech. (A) Group-averaged time course of BOLD activation for Speech (red) and Reversed (green) in left and right aSTS. ROIs were defined by *Speech* versus *SCN* (*P* < 0.001, uncorrected), (B) Half-maximum decay time of the BOLD response for speech and reversed speech. Bars denote group average, error bars represent 1 standard error of the mean. In similar fashion to pSTS results (Fig. 5), no significant difference was found between speech and reversed speech decay times, (C) Half-maximum decay times are plotted for speech against reversed speech in each participant. Dots under the gray line (*x* > *y*) are ones where reversed speech decays faster than speech. This is evident only in a few subjects in left aSTS, again, similarly to pSTS. aSTS, anterior superior temporal sulcus; ROIs, region of interests; SCN, signal correlated noise; pSTS, posterior superior temporal sulcus.

**Figure S2.** Reversed speech removes activation in language regions regardless of *P*-value. Axial slices of four individual participants, depicting significant response for *Speech* versus *SCN* contrast (left column) and *Speech* versus *Reversed* speech contrast (right columns), under different threshold levels. Notice that even for less stringent thresholds, *Speech* versus *Reversed* speech fails to detect activation in language regions which are readily picked up by the *Speech* versus *SCN* contrast. SCN, signal correlated noise.

**Figure S3.** Time courses of BOLD responses in three individual participants. Data were collected from ROI voxels identified by Speech > SCN contrast (*P* < 0.001, uncorrected). Activations are normalized to the baseline of rest signal level. Red = Speech, green = Reversed speech, blue = SCN. BOLD responses for speech and reversed speech rise similarly during initial stimulus presentation, but then decay more rapidly in the reversed condition. This effect was found mainly in the left IFG. ROI, region of interest; SCN, signal correlated noise; IFG, inferior frontal gyrus.

**Figure S4.** Group analysis results. Group activation patterns shown for *Speech* versus *SCN* (left panel) and S*peech* versus *Reversed* (right panel), displayed on the left hemisphere (*n* = 12, *P* < 0.001, uncorrected). Activations are shown in color rendered unto the SPM5 single subject brain template. Notice the absence of activation in left IFG for *Speech* versus *Reversed*, demonstrating that the difference in efficiency between the baselines is evident even at the group level. SCN, signal correlated noise; IFG, inferior frontal gyrus.
