# Supplementary figures and images for: Do not throw out the baby with the bath water: choosing an effective baseline for a functional localizer of speech processing
Source: Brain Behav. 2013 Feb 17;3(3):211–22. doi: 10.1002/brb3.129 (PMC3683281; doi:10.1002/brb3.129)

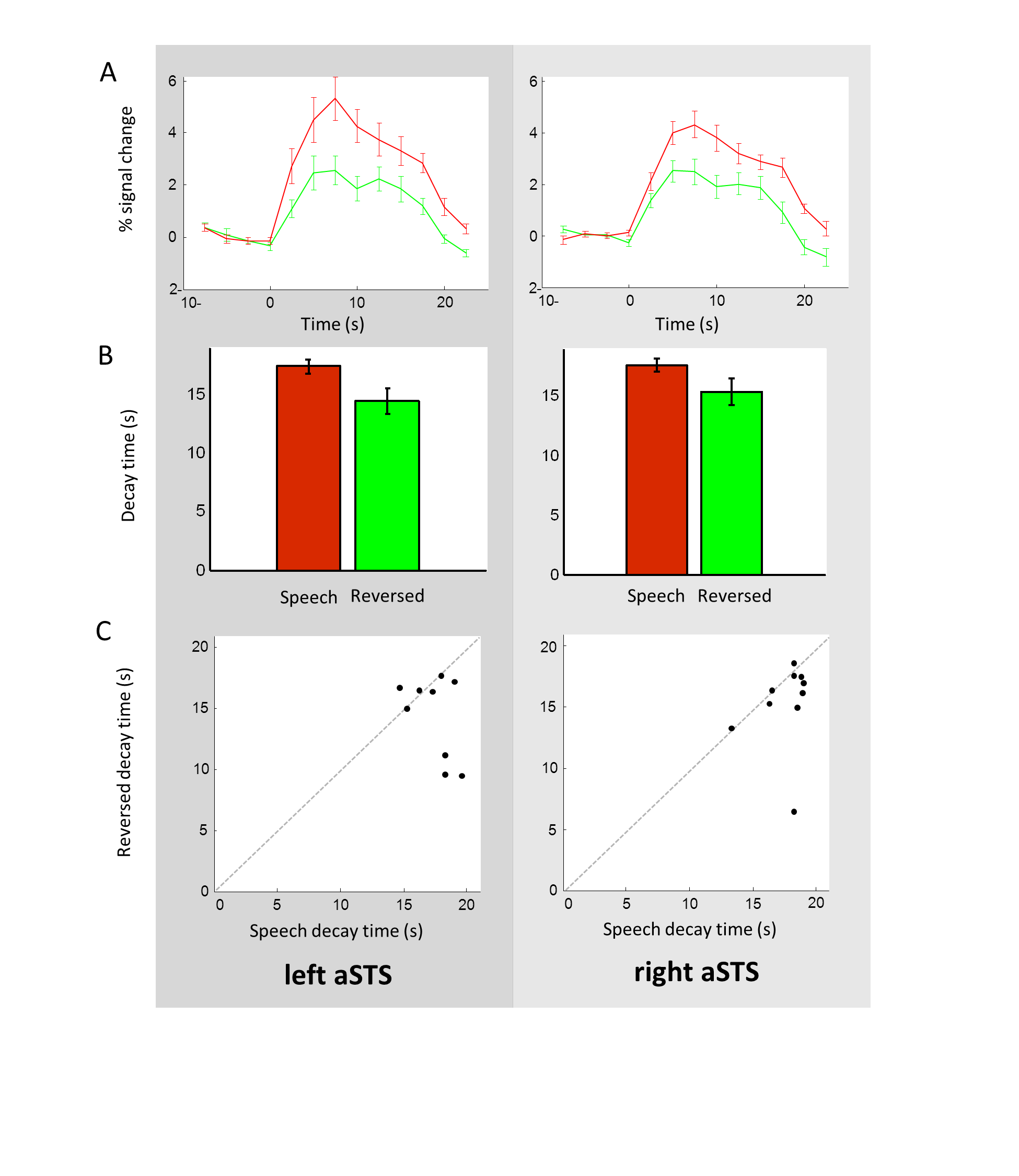

Supplement: Supplementary file 1 [file brb30003-0211-SD1.tif]

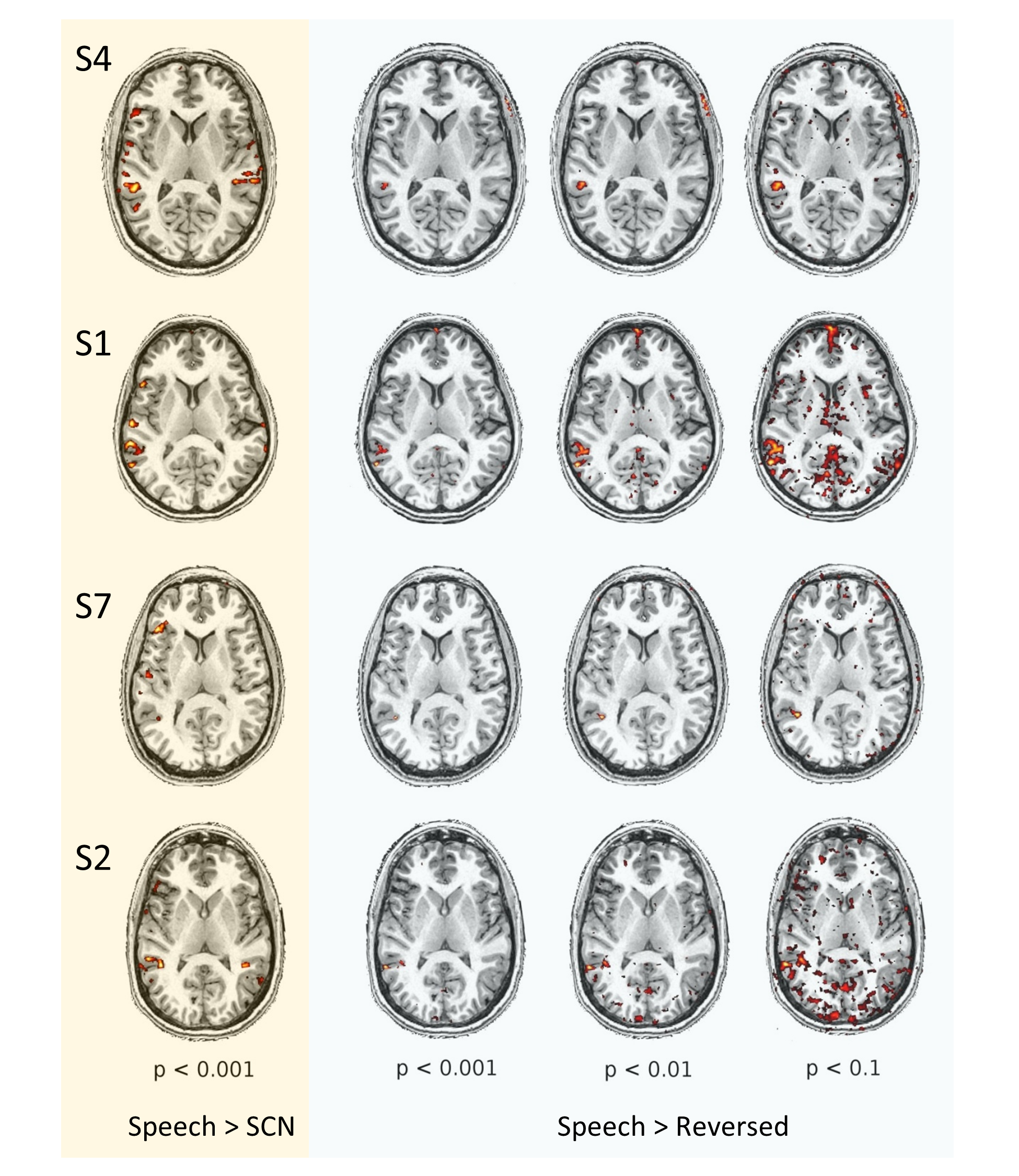

Supplement: Supplementary file 2 [file brb30003-0211-SD2.tif]

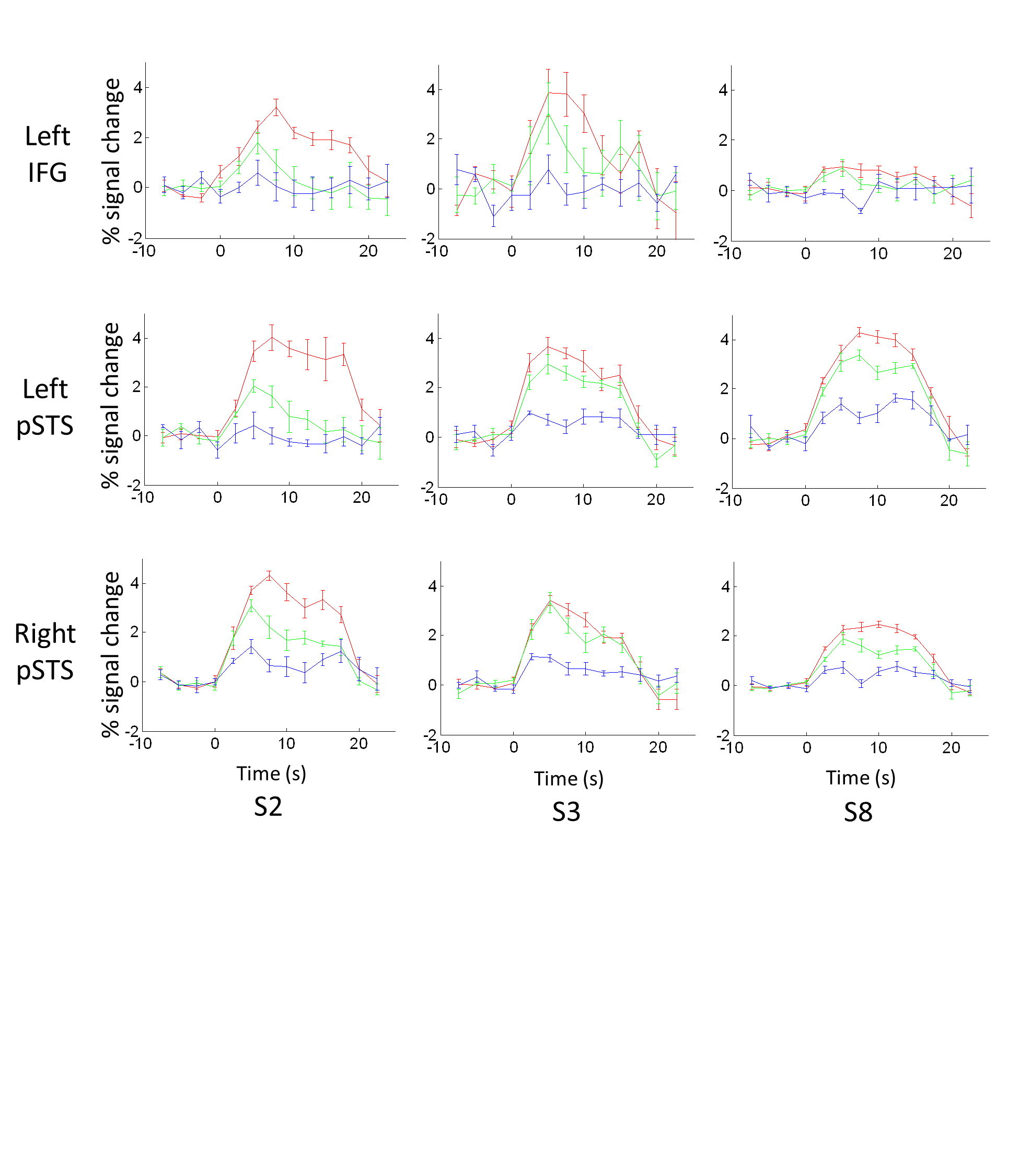

Supplement: Supplementary file 3 [file brb30003-0211-SD3.tif]

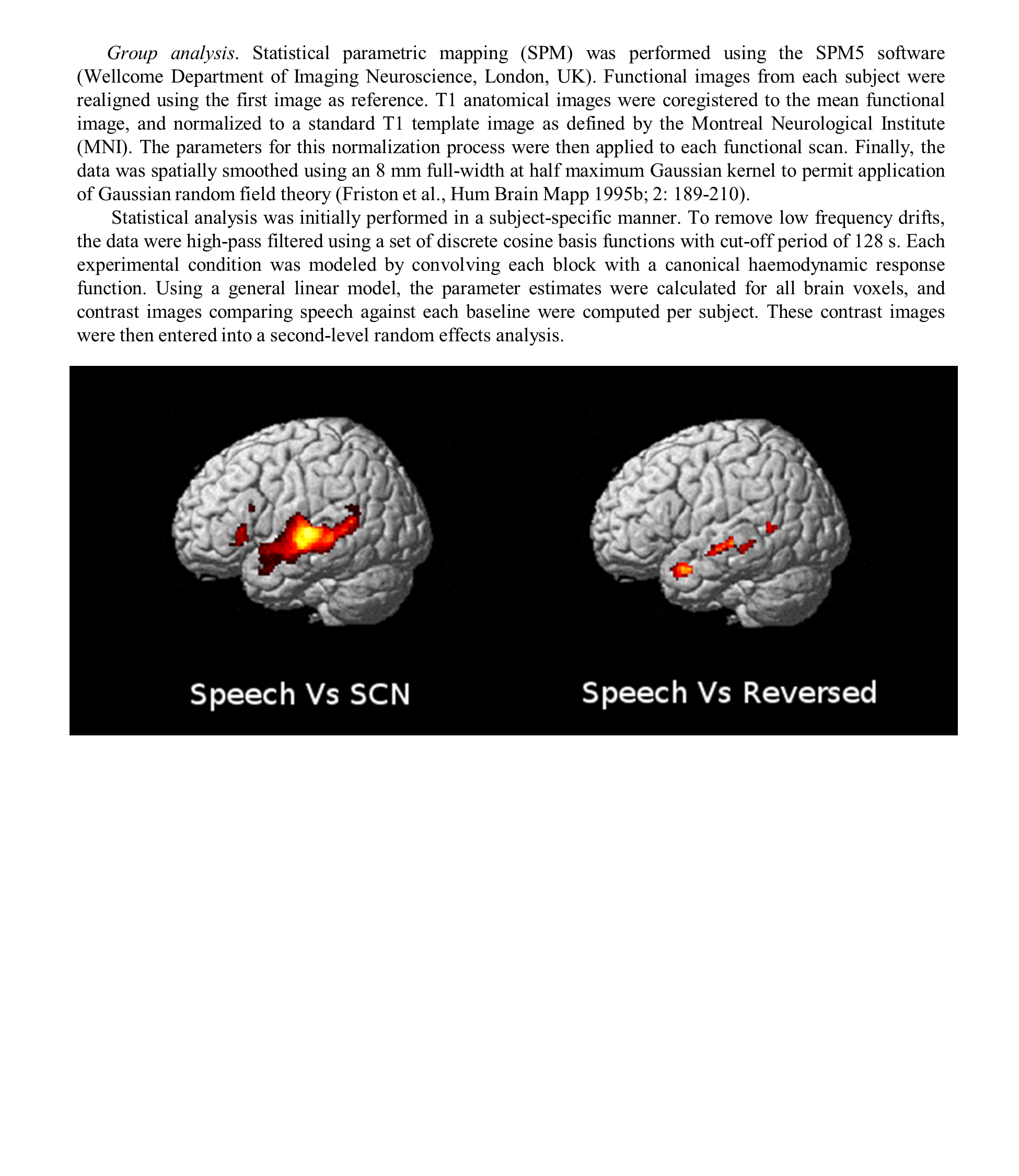

Supplement: Supplementary file 4 [file brb30003-0211-SD4.tif]
